# Caesium diuranium hexa­telluride

**DOI:** 10.1107/S1600536812038512

**Published:** 2012-09-19

**Authors:** Adel Mesbah, James A. Ibers

**Affiliations:** a Department of Chemistry, Northwestern University, 2145 Sheridan Road, Evanston, IL 60208, USA

## Abstract

Single crystals of CsU_2_Te_6_ were synthesized from the reaction of U, Te, and Cs_2_Te_3_ at 1273 K. CsU_2_Te_6_ crystallizes in the space group *Cmcm* in the CsTh_2_Te_6_ structure type. The asymmetric unit comprises one U (site symmetry *m*2*m*), one Cs (*m*2*m*; half-occupancy) and two Te atoms (*m*.. and *m*2*m*). The structure of CsU_2_Te_6_ consists of infinite [U_2_Te_6_] layers perpendicular to [010] separated by Cs atoms. There are infinite Te—Te—Te linear chains along [001].

## Related literature

For related structures, see: Narducci & Ibers (1998 ▶); Chan *et al.* (2004 ▶); Bugaris *et al.* (2010 ▶); Choi *et al.* (1998 ▶); Cody & Ibers (1996 ▶); Mizoguchi *et al.* (2006 ▶); Tougait *et al.* (1997 ▶); Krönert & Plieth (1965 ▶); Wu *et al.* (1997 ▶). For synthetic details, see: Bugaris & Ibers (2008 ▶); Haneveld & Jellinek (1969 ▶). For standardization of structural data, see: Gelato & Parthé (1987 ▶).

## Experimental

### 

#### Crystal data

CsU_2_Te_6_
*M* *_r_* = 1374.57
Orthorhombic, 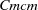

*a* = 4.2129 (2) Å
*b* = 25.6317 (11) Å
*c* = 6.0385 (2) Å
*V* = 652.06 (5) Å^3^
*Z* = 2
Mo *K*α radiation
μ = 40.65 mm^−1^
*T* = 100 K
0.21 × 0.03 × 0.02 mm

#### Data collection

Bruker APEXII CCD diffractometer
Absorption correction: numerical face-indexed (*SADABS*; Sheldrick, 2008*a* ▶) *T*_min_ = 0.043, *T*_max_ = 0.482
5645 measured reflections
611 independent reflections
574 reflections with *I* > 2σ(*I*)
*R*_int_ = 0.032

#### Refinement

*R*[*F* ^2^ > 2σ(*F* ^2^)] = 0.022
*wR*(*F* ^2^) = 0.053
*S* = 1.22
611 reflections
19 parameters
Δρ_max_ = 3.89 e Å^−3^
Δρ_min_ = −1.87 e Å^−3^

### 

Data collection: *APEX2* (Bruker, 2009 ▶); cell refinement: *SAINT* (Bruker, 2009 ▶); data reduction: *SAINT*; program(s) used to solve structure: *SHELXS97* (Sheldrick, 2008*b* ▶); program(s) used to refine structure: *SHELXL97* (Sheldrick, 2008*b* ▶); molecular graphics: *CrystalMaker* (Palmer, 2009 ▶); software used to prepare material for publication: *SHELXL97*.

## Supplementary Material

Crystal structure: contains datablock(s) I, global. DOI: 10.1107/S1600536812038512/br2209sup1.cif

Structure factors: contains datablock(s) I. DOI: 10.1107/S1600536812038512/br2209Isup2.hkl

Additional supplementary materials:  crystallographic information; 3D view; checkCIF report

## Figures and Tables

**Fig. 1.**
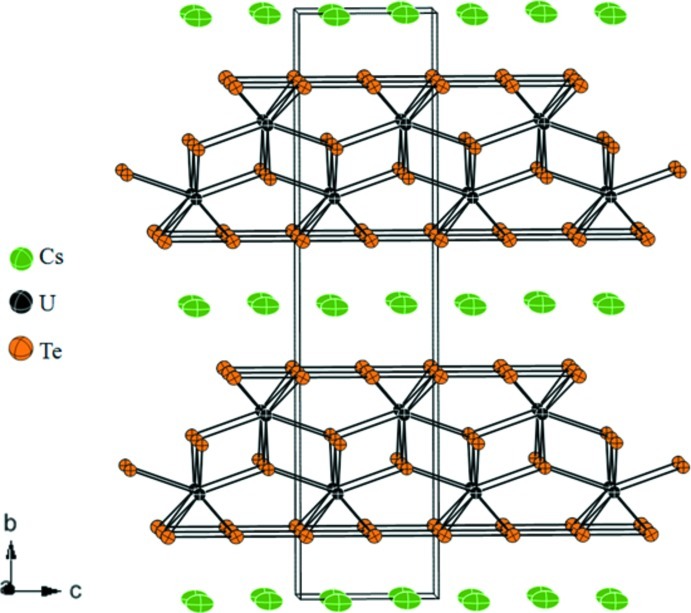
Structure of CsU2Te6 viewed approximately down [100]. Displacement ellipsoids are drawn at the 95% probability level.

## supplementary crystallographic information

### Comment

CsU_2_Te_6_ (Figure 1) belongs to the AAn_2_Q_6_ (A= K, Rb, Cs, or Tl; An = U, Th, or Np; Q = S, Se, or Te) family. Compounds in this family crystallize in two different structures types: CsTh_2_Te_6_ (Cody & Ibers, 1996) (space group *Cmcm*) and KTh_2_Se_6_ (Choi *et al.*, 1998; Wu *et al.*, 1997) (space group *Immm*). Both structure types have AnQ_3_ layers intercalated with A atoms. The difference between the two structure types is that each successive AnQ_3_ layer in the *Cmcm* structure type is shifted by a/2, whereas each successive AnQ_3_ layer in the *Immm* structure type is shifted by (a + b)/2 (Mizoguchi *et al.*, 2006). The AnQ_3_ layers are analogous to those in the structure of ZrSe_3_ (Krönert & Plieth, 1965). The CsTh_2_Te_6_ structure type is adopted by KTh_2_Te_6_ (Wu *et al.*, 1997) and Tl_1.12_UTe_6_ (Tougait *et al.*, 1997). The KTh_2_Se_6_ structure type is adopted by RbTh_2_Se_6_ (Choi *et al.*, 1998), K_0.91_U_1.79_S_6_ (Mizoguchi *et al.*, 2006), KU_2_Se_6_ (Chan *et al.*, 2004; Mizoguchi *et al.*, 2006), CsU_2_Se_6_ (Choi *et al.*, 1998), CsTh_2_Se_6_, Rb_0.85_U_1.74_S_6_, RbU_2_Se_6_, Cs_0.88_(La_0.68_U_1.32_)Se_6_, KNp_2_Se_6_, CsNp_2_Se_6_, and TlU_2_Se_6_ (Bugaris *et al.*, 2010). The structures of the two last compounds are modulated and were refined in 5a *x* 5b *x* 5c and 4a *x* 4b superlattices, respectively.

### Experimental

Black needles of CsU_2_Te_6_ were obtained by direct combination of ^238^U (30 mg, 12.9 mmol), Te (20.9 mg, 16.4 mmol, Aldrich, 99.8%) and Cs_2_Te_3_ (24.6 mg, 37.9 mmol). Cs_2_Te_3_ was prepared by the stoichiometric reaction of Cs (Alfa Aesar, 99.8%) and Te in liquid NH_3_ at 194 K. U powder obtained by hydridization and decomposition of turnings (depleted, ORNL) by heating under vacuum, in a modification (Bugaris & Ibers, 2008) of a previous literature method (Haneveld & Jellinek, 1969). The starting reagents were loaded in a carbon-coated fused-silica tube under an Ar atmosphere in a glove box, then evacuated to 10 ^-4^ Torr, and flame sealed. The tube was placed in computer-controlled furnace, heated to 1273 K in 48 h, held there for 4 h, cooled to 1223 K in 12 h and kept there for 8 d, then cooled to 293 K at 3 K/ h. Black needles were selected and analyzed by EDX and showed the formation of Cs:U:Te in a 1:2:6 ratio. The yield, based on U, was about 15% of the product.

### Refinement

The highest peak (3.9 e^-^ Å^-3^) is 0.76 Å from atom Te1 and the deepest hole (1.9 e^-^ Å^-3^) is 0.78 Å from atom U1. These should be compared with the height of 225 e^-^ Å^-3^ of atom Te(1) in an electron density map.

### Crystal data

**Table:**

CsU_2_Te_6_	*F*(000) = 1102
*M**_r_* = 1374.57	*D*_x_ = 7.001 Mg m^−^^3^
Orthorhombic, *C**m**c**m*	Mo *K*α radiation, λ = 0.71073 Å
Hall symbol: -C 2c 2	Cell parameters from 2738 reflections
*a* = 4.2129 (2) Å	θ = 6.4–60.9°
*b* = 25.6317 (11) Å	µ = 40.65 mm^−^^1^
*c* = 6.0385 (2) Å	*T* = 100 K
*V* = 652.06 (5) Å^3^	Needle, black
*Z* = 2	0.21 × 0.03 × 0.02 mm

### Data collection

**Table:**

Bruker APEXII CCD diffractometer	611 independent reflections
Radiation source: fine-focus sealed tube	574 reflections with *I* > 2σ(*I*)
Graphite monochromator	*R*_int_ = 0.032
φ and ω scans	θ_max_ = 30.7°, θ_min_ = 3.2°
Absorption correction: numerical face-indexed (*SADABS*; Sheldrick, 2008*a*)	*h* = −5→3
*T*_min_ = 0.043, *T*_max_ = 0.482	*k* = −36→36
5645 measured reflections	*l* = −8→8

### Refinement

**Table:**

Refinement on *F*^2^	0 restraints
Least-squares matrix: full	Primary atom site location: structure-invariant direct methods
*R*[*F*^2^ > 2σ(*F*^2^)] = 0.022	Secondary atom site location: difference Fourier map
*wR*(*F*^2^) = 0.053	[1.00000]/[σ^2^(*F*_o_^2^) + (0.0298*F*_o_^2^)^2^]
*S* = 1.22	(Δ/σ)_max_ = 0.002
611 reflections	Δρ_max_ = 3.89 e Å^−^^3^
19 parameters	Δρ_min_ = −1.87 e Å^−^^3^

### Special details

**Table:**

Geometry. All e.s.d.'s (except the e.s.d. in the dihedral angle between two l.s. planes) are estimated using the full covariance matrix. The cell e.s.d.'s are taken into account individually in the estimation of e.s.d.'s in distances, angles and torsion angles; correlations between e.s.d.'s in cell parameters are only used when they are defined by crystal symmetry. An approximate (isotropic) treatment of cell e.s.d.'s is used for estimating e.s.d.'s involving l.s. planes.
Refinement. Refinement of *F*^2^ against ALL reflections. The weighted *R*-factor *wR* and goodness of fit *S* are based on *F*^2^, conventional *R*-factors *R* are based on *F*, with *F* set to zero for negative *F*^2^. The threshold expression of *F*^2^ > σ(*F*^2^) is used only for calculating *R*-factors(gt) *etc*. and is not relevant to the choice of reflections for refinement. *R*-factors based on *F*^2^ are statistically about twice as large as those based on *F*, and *R*- factors based on ALL data will be even larger.

### Fractional atomic coordinates and isotropic or equivalent isotropic displacement parameters (Å^2^)

**Table:**

	*x*	*y*	*z*	*U*_iso_*/*U*_eq_	Occ. (<1)
U1	0.0000	0.685078 (14)	0.2500	0.00916 (11)	
Cs1	0.0000	0.49825 (7)	0.2500	0.0556 (7)	0.50
Te1	0.0000	0.116539 (18)	0.00249 (7)	0.01098 (13)	
Te2	0.0000	0.27322 (2)	0.2500	0.00877 (14)	

### Atomic displacement parameters (Å^2^)

**Table:**

	*U*^11^	*U*^22^	*U*^33^	*U*^12^	*U*^13^	*U*^23^
U1	0.0072 (2)	0.01045 (17)	0.00982 (17)	0.000	0.000	0.000
Cs1	0.100 (2)	0.0194 (9)	0.0475 (12)	0.000	0.000	0.000
Te1	0.0087 (3)	0.0118 (2)	0.0125 (2)	0.000	0.000	−0.00052 (15)
Te2	0.0079 (3)	0.0090 (3)	0.0095 (3)	0.000	0.000	0.000

### Geometric parameters (Å, º)

**Table:**

U1—Te2^i^	3.0890 (5)	Cs1—Te1^iii^	3.9830 (15)
U1—Te2^ii^	3.0890 (5)	Cs1—Te1^ii^	3.9830 (15)
U1—Te1^ii^	3.1237 (4)	Cs1—Cs1^xi^	4.2129 (2)
U1—Te1^iii^	3.1237 (4)	Cs1—Cs1^xii^	4.2129 (2)
U1—Te1^iv^	3.1237 (4)	Te1—Te1^xiii^	2.9892 (8)
U1—Te1^i^	3.1237 (4)	Te1—Te1^xiv^	3.0493 (8)
U1—Te2^v^	3.2028 (3)	Te1—U1^xv^	3.1237 (4)
U1—Te2^vi^	3.2028 (3)	Te1—U1^xvi^	3.1237 (4)
U1—Cs1	4.7888 (19)	Te1—Cs1^vii^	3.9265 (14)
Cs1—Cs1^v^	3.0206 (2)	Te1—Cs1^ix^	3.9265 (14)
Cs1—Cs1^vi^	3.0206 (2)	Te1—Cs1^xvi^	3.9830 (15)
Cs1—Te1^vii^	3.9266 (14)	Te1—Cs1^xv^	3.9830 (15)
Cs1—Te1^viii^	3.9266 (14)	Te2—U1^xvi^	3.0889 (5)
Cs1—Te1^ix^	3.9266 (14)	Te2—U1^xv^	3.0889 (5)
Cs1—Te1^x^	3.9266 (14)	Te2—U1^v^	3.2028 (3)
Cs1—Te1^iv^	3.9830 (15)	Te2—U1^vi^	3.2028 (3)
Cs1—Te1^i^	3.9830 (15)		
			
Te2^i^—U1—Te2^ii^	85.989 (18)	Te1^ix^—Cs1—Te1^iii^	178.90 (3)
Te2^i^—U1—Te1^ii^	150.600 (9)	Te1^x^—Cs1—Te1^iii^	135.105 (5)
Te2^ii^—U1—Te1^ii^	87.220 (10)	Te1^iv^—Cs1—Te1^iii^	63.86 (3)
Te2^i^—U1—Te1^iii^	150.600 (9)	Te1^i^—Cs1—Te1^iii^	80.85 (4)
Te2^ii^—U1—Te1^iii^	87.220 (10)	Cs1^v^—Cs1—Te1^ii^	110.64 (7)
Te1^ii^—U1—Te1^iii^	57.172 (15)	Cs1^vi^—Cs1—Te1^ii^	66.56 (5)
Te2^i^—U1—Te1^iv^	87.220 (10)	Te1^vii^—Cs1—Te1^ii^	98.104 (9)
Te2^ii^—U1—Te1^iv^	150.600 (9)	Te1^viii^—Cs1—Te1^ii^	115.619 (7)
Te1^ii^—U1—Te1^iv^	111.555 (18)	Te1^ix^—Cs1—Te1^ii^	135.105 (5)
Te1^iii^—U1—Te1^iv^	84.808 (13)	Te1^x^—Cs1—Te1^ii^	178.90 (3)
Te2^i^—U1—Te1^i^	87.220 (10)	Te1^iv^—Cs1—Te1^ii^	80.85 (4)
Te2^ii^—U1—Te1^i^	150.600 (9)	Te1^i^—Cs1—Te1^ii^	63.86 (3)
Te1^ii^—U1—Te1^i^	84.808 (13)	Te1^iii^—Cs1—Te1^ii^	44.08 (2)
Te1^iii^—U1—Te1^i^	111.555 (18)	Cs1^v^—Cs1—Cs1^xi^	90.0
Te1^iv^—U1—Te1^i^	57.172 (15)	Cs1^vi^—Cs1—Cs1^xi^	90.0
Te2^i^—U1—Te2^v^	75.873 (8)	Te1^vii^—Cs1—Cs1^xi^	57.556 (13)
Te2^ii^—U1—Te2^v^	75.873 (8)	Te1^viii^—Cs1—Cs1^xi^	57.556 (13)
Te1^ii^—U1—Te2^v^	129.697 (9)	Te1^ix^—Cs1—Cs1^xi^	122.442 (13)
Te1^iii^—U1—Te2^v^	74.730 (11)	Te1^x^—Cs1—Cs1^xi^	122.442 (13)
Te1^iv^—U1—Te2^v^	74.730 (11)	Te1^iv^—Cs1—Cs1^xi^	121.930 (13)
Te1^i^—U1—Te2^v^	129.697 (9)	Te1^i^—Cs1—Cs1^xi^	121.930 (13)
Te2^i^—U1—Te2^vi^	75.873 (8)	Te1^iii^—Cs1—Cs1^xi^	58.072 (13)
Te2^ii^—U1—Te2^vi^	75.873 (8)	Te1^ii^—Cs1—Cs1^xi^	58.072 (13)
Te1^ii^—U1—Te2^vi^	74.730 (11)	Cs1^v^—Cs1—Cs1^xii^	90.0
Te1^iii^—U1—Te2^vi^	129.697 (9)	Cs1^vi^—Cs1—Cs1^xii^	90.0
Te1^iv^—U1—Te2^vi^	129.697 (9)	Te1^vii^—Cs1—Cs1^xii^	122.442 (13)
Te1^i^—U1—Te2^vi^	74.730 (11)	Te1^viii^—Cs1—Cs1^xii^	122.442 (13)
Te2^v^—U1—Te2^vi^	141.01 (2)	Te1^ix^—Cs1—Cs1^xii^	57.556 (13)
Te2^i^—U1—Cs1	137.005 (9)	Te1^x^—Cs1—Cs1^xii^	57.556 (13)
Te2^ii^—U1—Cs1	137.005 (9)	Te1^iv^—Cs1—Cs1^xii^	58.072 (13)
Te1^ii^—U1—Cs1	55.777 (9)	Te1^i^—Cs1—Cs1^xii^	58.072 (13)
Te1^iii^—U1—Cs1	55.777 (9)	Te1^iii^—Cs1—Cs1^xii^	121.930 (13)
Te1^iv^—U1—Cs1	55.777 (9)	Te1^ii^—Cs1—Cs1^xii^	121.930 (13)
Te1^i^—U1—Cs1	55.777 (9)	Cs1^xi^—Cs1—Cs1^xii^	180.0
Te2^v^—U1—Cs1	109.494 (12)	Te1^xiii^—Te1—Te1^xiv^	180.00 (3)
Te2^vi^—U1—Cs1	109.494 (12)	Te1^xiii^—Te1—U1^xv^	61.414 (8)
Cs1^v^—Cs1—Cs1^vi^	176.59 (14)	Te1^xiv^—Te1—U1^xv^	118.586 (8)
Cs1^v^—Cs1—Te1^vii^	114.23 (7)	Te1^xiii^—Te1—U1^xvi^	61.414 (8)
Cs1^vi^—Cs1—Te1^vii^	68.54 (5)	Te1^xiv^—Te1—U1^xvi^	118.586 (8)
Cs1^v^—Cs1—Te1^viii^	68.54 (5)	U1^xv^—Te1—U1^xvi^	84.806 (13)
Cs1^vi^—Cs1—Te1^viii^	114.23 (7)	Te1^xiii^—Te1—Cs1^vii^	112.848 (11)
Te1^vii^—Cs1—Te1^viii^	45.70 (2)	Te1^xiv^—Te1—Cs1^vii^	67.152 (11)
Cs1^v^—Cs1—Te1^ix^	114.23 (7)	U1^xv^—Te1—Cs1^vii^	165.69 (2)
Cs1^vi^—Cs1—Te1^ix^	68.54 (5)	U1^xvi^—Te1—Cs1^vii^	104.208 (12)
Te1^vii^—Cs1—Te1^ix^	64.89 (3)	Te1^xiii^—Te1—Cs1^ix^	112.848 (11)
Te1^viii^—Cs1—Te1^ix^	82.94 (4)	Te1^xiv^—Te1—Cs1^ix^	67.152 (11)
Cs1^v^—Cs1—Te1^x^	68.54 (5)	U1^xv^—Te1—Cs1^ix^	104.208 (12)
Cs1^vi^—Cs1—Te1^x^	114.23 (7)	U1^xvi^—Te1—Cs1^ix^	165.69 (2)
Te1^vii^—Cs1—Te1^x^	82.94 (4)	Cs1^vii^—Te1—Cs1^ix^	64.89 (3)
Te1^viii^—Cs1—Te1^x^	64.89 (3)	Te1^xiii^—Te1—Cs1^xvi^	67.961 (10)
Te1^ix^—Cs1—Te1^x^	45.70 (2)	Te1^xiv^—Te1—Cs1^xvi^	112.039 (10)
Cs1^v^—Cs1—Te1^iv^	66.56 (5)	U1^xv^—Te1—Cs1^xvi^	127.246 (13)
Cs1^vi^—Cs1—Te1^iv^	110.64 (7)	U1^xvi^—Te1—Cs1^xvi^	83.80 (2)
Te1^vii^—Cs1—Te1^iv^	178.90 (3)	Cs1^vii^—Te1—Cs1^xvi^	44.895 (5)
Te1^viii^—Cs1—Te1^iv^	135.105 (5)	Cs1^ix^—Te1—Cs1^xvi^	81.896 (9)
Te1^ix^—Cs1—Te1^iv^	115.619 (7)	Te1^xiii^—Te1—Cs1^xv^	67.961 (10)
Te1^x^—Cs1—Te1^iv^	98.104 (9)	Te1^xiv^—Te1—Cs1^xv^	112.039 (10)
Cs1^v^—Cs1—Te1^i^	110.64 (7)	U1^xv^—Te1—Cs1^xv^	83.80 (2)
Cs1^vi^—Cs1—Te1^i^	66.56 (5)	U1^xvi^—Te1—Cs1^xv^	127.246 (13)
Te1^vii^—Cs1—Te1^i^	135.105 (5)	Cs1^vii^—Te1—Cs1^xv^	81.896 (9)
Te1^viii^—Cs1—Te1^i^	178.90 (3)	Cs1^ix^—Te1—Cs1^xv^	44.895 (5)
Te1^ix^—Cs1—Te1^i^	98.104 (9)	Cs1^xvi^—Te1—Cs1^xv^	63.86 (3)
Te1^x^—Cs1—Te1^i^	115.619 (7)	U1^xvi^—Te2—U1^xv^	85.992 (18)
Te1^iv^—Cs1—Te1^i^	44.08 (2)	U1^xvi^—Te2—U1^v^	104.126 (8)
Cs1^v^—Cs1—Te1^iii^	66.56 (5)	U1^xv^—Te2—U1^v^	104.126 (8)
Cs1^vi^—Cs1—Te1^iii^	110.64 (7)	U1^xvi^—Te2—U1^vi^	104.126 (8)
Te1^vii^—Cs1—Te1^iii^	115.619 (7)	U1^xv^—Te2—U1^vi^	104.126 (8)
Te1^viii^—Cs1—Te1^iii^	98.104 (9)	U1^v^—Te2—U1^vi^	141.01 (2)

Symmetry codes: (i) *x*+1/2, *y*+1/2, *z*; (ii) *x*−1/2, *y*+1/2, *z*; (iii) *x*−1/2, *y*+1/2, −*z*+1/2; (iv) *x*+1/2, *y*+1/2, −*z*+1/2; (v) −*x*, −*y*+1, −*z*+1; (vi) −*x*, −*y*+1, −*z*; (vii) −*x*−1/2, −*y*+1/2, −*z*; (viii) −*x*−1/2, −*y*+1/2, *z*+1/2; (ix) −*x*+1/2, −*y*+1/2, −*z*; (x) −*x*+1/2, −*y*+1/2, *z*+1/2; (xi) *x*−1, *y*, *z*; (xii) *x*+1, *y*, *z*; (xiii) *x*, *y*, −*z*+1/2; (xiv) *x*, *y*, −*z*−1/2; (xv) *x*+1/2, *y*−1/2, *z*; (xvi) *x*−1/2, *y*−1/2, *z*.
